# Vascular Cognitive Impairment: Information from Animal Models on the Pathogenic Mechanisms of Cognitive Deficits

**DOI:** 10.3390/ijms20102405

**Published:** 2019-05-15

**Authors:** Jakub Hort, Martin Vališ, Kamil Kuča, Francesco Angelucci

**Affiliations:** 1 Memory Clinic, Department of Neurology, 2nd Faculty of Medicine, Charles University and Motol University Hospital, 150 06 Prague, Czech Republic; jakub.hort@gmail.com; 2 International Clinical Research Centre, St. Anne’s University Hospital, 656 91 Brno, Czech Republic; 3 Department of Neurology, University Hospital Hradec Králové, Charles University in Prague, Faculty of Medicine in Hradec Králové, Sokolská Street 581, 500 05 Hradec Králové, Czech Republic; valismar@seznam.cz; 4 Department of Chemistry, Faculty of Science, University of Hradec Kralove, 500 05 Hradec Kralove, Czech Republic; kamil.kuca@uhk.cz

**Keywords:** vascular cognitive impairment, oxidative stress, neuroinflammation, glial cells, IGF-1

## Abstract

Vascular cognitive impairment (VCI) is the second most common cause of cognitive deficit after Alzheimer’s disease. Since VCI patients represent an important target population for prevention, an ongoing effort has been made to elucidate the pathogenesis of this disorder. In this review, we summarize the information from animal models on the molecular changes that occur in the brain during a cerebral vascular insult and ultimately lead to cognitive deficits in VCI. Animal models cannot effectively represent the complex clinical picture of VCI in humans. Nonetheless, they allow some understanding of the important molecular mechanisms leading to cognitive deficits. VCI may be caused by various mechanisms and metabolic pathways. The pathological mechanisms, in terms of cognitive deficits, may span from oxidative stress to vascular clearance of toxic waste products (such as amyloid beta) and from neuroinflammation to impaired function of microglia, astrocytes, pericytes, and endothelial cells. Impaired production of elements of the immune response, such as cytokines, and vascular factors, such as insulin-like growth factor 1 (IGF-1), may also affect cognitive functions. No single event could be seen as being the unique cause of cognitive deficits in VCI. These events are interconnected, and may produce cascade effects resulting in cognitive impairment.

## 1. Vascular Cognitive Impairment (VCI)

Vascular cognitive impairment (VCI) [1] comprises both subjects with dementia and mild cognitive impairment (MCI). The symptoms of VCI include the disturbance of specific cognitive functions with various degrees of severity [2]. VCI is recognized to be the second most common cause of cognitive deficit after Alzheimer’s disease (AD). However, a comorbidity between these pathologies is frequent and characterized by synergistic processes leading to cognitive impairments [3]. This is the reason why subjects with VCI are often classified as AD, and thus VCI’s ranking as the cause of cognitive deficits may vary from second to fourth [4]. Various biomarkers have been used to increase the certainty of diagnosis underlying pathological processes [5,6,7,8,9,10].

VCI is a general term that includes a group of cognitive disorders attributable to a pathological state of the cerebral vascular system [11]. In the brain, VCI is characterized by the atrophy of gray matter and hemispheric white matter lesions [12], but may include also subcortical vascular lesions in, for example, the thalamus. The main cognitive deficits usually involve memory processes, speed of processing, and executive functions [2]. VCI types are classified according to clinical characteristics, and include vascular mild cognitive impairment (VMCI), vascular dementia (VaD), and mixed dementia (MD) associated with vascular dysfunction [13].

VCI prevalence is also age- and risk factor-related. In older people over 65 years, the prevalence of mild VCI is higher than that of VD [14]. Among other risk factors, the most relevant are those affecting the vascular system and include hypertension, hyperlipidemia, hyperuricemia, diabetes, cardiopathy, history of stroke, carotid plaque, and smoking [15].

The conceptualization of VCI has recently evolved with the adoption of VASCOG [16], VICCCS [13], and DSM-V [17] criteria. Subtypes of VCI are divided into mild VCI and major VCI (VaD) according to the level of VCI impairment, with operational criteria for the thresholds, and clinical and neuroimaging criteria to establish vascular etiology. These new criteria were adopted to overcome the limitations of those by DSM-IV and NINDS-AIREN [18] which, having memory impairment as necessary criterion for the diagnosis of VCI, often bias the diagnosis towards AD, and do not distinguish among deficits in different cognitive domains. In addition, the assessment of brain microvasculature impairment as a co-morbidity is becoming fundamental for staging cognitive decline in MCI and AD [8,19,20]. It was shown that subjects with normal functioning at the stage of MCI are more likely to progress to dementia if their Fazekas vascular score is higher [21].

Since VCI patients represent an important target population for prevention, great efforts have been made to elucidate the pathogenesis of this disorder.

## 2. Pathogenesis

Many studies have provided new insights into the causes of VCI. VCI is caused by decreased blood supply to the brain [11]. The affected brain regions undergo a neuronal tissue loss which compromises its structure and function and manifests as a cognitive deficit.

The causes of this reduced blood flow can be divided into three main groups: ischemic factors, hemorrhagic factors, and other factors affecting functional brain regions [22].

### 2.1. Ischemic Factors

Vessel obstruction can be the cause of VCI and dementia and, generally, with latency after the event. Vessel occlusions can be large, as in the case of ischemic stroke, or small, such as when caused by arteritis and arteriosclerosis of smaller vessels, where the term ‘small vessel dysfunction’ is more appropriate.

In the case of stroke, inflammatory mediators and amyloid deposition (cerebral amyloid angiopathy (CAA)) in the walls of vessels play an important role in the development of VCI [23,24]. The cognitive deficits after stroke generally occur in a shorter time frame (i.e., less than 1 year) as compared to other forms of VCI [13,25].

The damage caused by dysfunction of small vessels is slower to appear as it is the result of cortical and subcortical microinfarcts [26]. In this condition, the involved brain region is affected by a state of cerebral hypoperfusion which, in the long term, is responsible for the damage of white matter and for the insurgence of cognitive dysfunction [27,28]. These types of multiple infarctions and diffuse white matter lesions often appear in the lateral ventricle and subcortical structures, resulting in multiple cognitive domain impairment [26,27,28].

### 2.2. Hemorrhagic Factors

Vascular dysfunction and cognitive deficits can also occur after a cerebral hemorrhage [27,28]. Cognitive dysfunction can originate from an intracranial hemorrhage, caused by CAA [29,30] and resulting in brain edema or secondary ischemia due to mass lesion. Also, a subarachnoid hemorrhage [31], with involvement of hippocampal and frontotemporal regions [32], can result in VCI syndrome with visuospatial memory and language deficits [33]. In this case, it is believed that VCI is attributed to the impact of the subdural membrane on dural lymphatic drainage [34].

### 2.3. Other Factors

VCI can appear in comorbidity with other diseases, such as AD, synucleinopathy, or tauopathy, which often results from both vascular disorders and structural changes in the protein of brain tissue [22]. In addition, hypertension [35] and diabetes [3], which represent independent risk factors for AD, can be concomitant with VCI.

Lifestyle factors may also contribute to VCI, including smoking [36], vitamin deficits (vitamin E) [37], poor physical activity [38], and a low level of education. However, the contribution of these factors to cognitive deficits in VCI is, at present, controversial [36].

An improved understanding of the contribution of vascular burden to cognitive decline further originates from investigations on translational animal models [39].

## 3. Animal Models of VCI

Animal models for studying VCI comprise different models based on the pathogenic factors [40] exposed above (Table 1).

The most-used species are rodents [41], although other species can be also used once the efficacy of a particular treatment has been established in rodents. Thus, models using rabbits, monkeys, and dogs were also investigated before translation to clinical trials [41].

Stroke is usually mimicked by a transient (tMCAO) or permanent (pMCAO) middle cerebral artery occlusion [42]. Small vessel occlusion is also mimicked by tMCAO [44]. A condition of global hypoperfusion can be induced by bilateral carotid artery occlusion (BCAO) or bilateral carotid artery stenosis (BCAS) [43]. These rodents present with hypoxia and hypoperfusion of white matter, making this a reasonable model for VCI [45].

A cerebral hemorrhage can be induced by intracerebral (double or single) injection of blood or collagenase [46], or induced by a microballoon inserted into the brain [47].

A subarachnoid hemorrhage may also created by the injection of blood [50]. In addition, microhemorrhages can be induced with high spatiotemporal precision in rodent cortex by directly disrupting the walls of cortical microvessels using focused lasers [48].

VCI-relevant animal models can also result from genetically manipulated animals. The stroke-prone spontaneously hypertensive rats (SHR/SP) animal model is the most relevant model of hypertension in rodents, and was created by inbred strain manipulation [43].

Another genetic model in mice was obtained by knocking out the *Notch3* gene [41,43]. This genetic manipulation resembles the hereditary form of VD [51], where the cerebrovascular alterations are similar to those observed in human autosomal dominant arteriopathy with subcortical infarcts and leukoencephalopathy (CADASIL) [52]. Other approaches to induce VCI in animals include mimicking risk factors such as diabetes, or by diet exposure to create risky conditions, such as hyperhomocysteinemia [49].

We review the current evidence obtained, from these models, for the pathogenesis of VCI.

## 4. Molecular Mechanism of VCI

Animal models may serve to investigate the molecular changes that occur in the brain during a cerebral vascular insult and that ultimately lead to the cognitive deficits of VCI.

Cerebral circulation assures an adequate delivery of blood to the brain. Hypoxia or chronic cerebral hypoperfusion may lead to an altered balance between the delivery of energy substrates and the clearance of metabolic waste in the neurovascular unit. The pathological mechanisms, in terms of cognitive deficits, may span from oxidative stress to vascular clearance of toxic waste products (such as Aβ), and from neuroinflammation to impaired function of microglia, astrocytes, and endothelial cells. Impaired production of elements of the immune response, such as cytokines, and vascular factors, such as insulin-like growth factor 1 (IGF-1), may also affect cognitive functions. At a molecular level, there are several lines of investigations depending on the supposed cause of the VCI.

### 4.1. Oxidative Stress

Oxidative stress is a condition of imbalance between free radicals and antioxidants [53,54] that can originate from vascular-related pathological states, including hypertension, diabetes, and arteriosclerosis [53]. In VCI, oxidative stress is considered to be a major contributing factor to the pathogenesis of cognitive deficits [55]. Moreover, it has been repetitively demonstrated that the excessive oxidation of proteins is also a common phenomenon in neurodegenerative disease and is correlated to cognitive deficit [56,57].

The molecular mechanism of oxidative stress-induced cognitive deficits has been investigated in animal models. In occlusion rat models (BCAO), it was shown that oxidative stress is characterized by the increased production of reactive oxygen species (ROS) [58,59] which are responsible for both cardiovascular pathophysiology and neurodegeneration [60,61]. In fact, ROS and reduced antioxidant defense may directly affect synaptic activity and neurotransmission in neurons, leading to cognitive dysfunction [62]. In the same models, it was also shown that the negative effects of ROS on memory processes could be counteracted by substances able to increase the gene expression of antioxidant proteins, such as nuclear factor erythroid 2 like 2 (NFE2L2), alcohol dehydrogenase 7 (ADH7), and glutathione peroxidase 2 (GPX2) and 3 (GPX3) [58,59].

Excessive ROS production during VCI is caused by the enzyme nicotinamide adenine dinucleotide phosphate (NADPH) oxidase [62]. NADPH oxidase is a multiunit enzyme that was discovered in neutrophils and is also present in vessel cells, particularly in cerebral blood vessels [62,63]. ROS derived from the enzyme NADPH oxidase are key pathogenic effectors of cerebrovascular dysregulation [63]. This might lead, in turn, to cognitive impairment via cellular dysfunction and cell death [62].

Supporting this notion, it has been shown that inhibition of NADPH oxidase activity can reduce cognitive impairment in BCAO rodent models of VCI [64,65]. In addition, it is likely that similar alterations may occur in human VCI. Data from subjects affected by VD demonstrated that the activity of antioxidant enzymes—such as superoxide dismutase (SOD), catalase (CAT), glutathione peroxidase (GPx), glutathione reductase (GR), and heme oxygenase/biliverdin reductase—is decreased [55,66]. Furthermore, reduced antioxidant enzyme levels in blood samples from VD patients have also been reported [67,68].

Taken together, these data suggest that oxidative stress, through the production of ROS, may induce cognitive deficits in humans. From this perspective, the targeting of such genes is an adoptable therapy to treat ischemic-induced cognitive deficits.

### 4.2. Neuroinflammation and Activation of Microglia

Oxidative stress is also the cause of inflammatory processes at the neurovascular unit. Thus, it is plausible that inflammation may also be involved in the pathophysiology of VCI [53,69].

Studies in rat models of BCAO have demonstrated that a condition of hypoxia/ischemia triggers microglia to release metalloproteinases (MMPs), which can damage the blood–brain barrier (BBB) and disrupt myelinated fibers [70,71]. In addition, neuroinflammation after BCAO can induce cognitive dysfunction through the release of proinflammatory cytokines and ROS by the activated microglia [72].

Chronic microglial activation can be responsible for the pathogenesis of different forms of dementia [73]. It has been shown, in BCAO models, that inflammatory-related microglia is associated with cognitive impairment [74]. This mechanism seems to be related to the activation of receptors of advanced glycation end products (RAGE) which are present on both microglia and neurons [59,75]. RAGE activation turns on nuclear factor kappa B (NF-κB), which is a transcription factor that controls several proinflammatory genes.

The release of cytokines, such as interleukin (IL)-6 and tumor necrosis factor (TNF)-α, by microglia play important roles in the pathogenesis of dementia [73]. It has been hypothesized that this pathway is also activated in AD [76]. In animal models of stroke and in humans, IL-6 is increased in serum and cerebrospinal fluid (CSF) after stroke [77,78]. In addition, it was shown that elevated levels of IL-6 contribute to the insurgence of dementia in patients with vascular risk factors [79]. Accordingly, patients with VD also show a high level of serum IL-6 [80], which points to the inflammatory component in the development of VCI.

### 4.3. Astrocytes

In the past years, the role of astrocytes as fine-tune regulators of neuronal activity have been thoroughly studied. Nevertheless, recent evidence suggests that astrocytes may be key elements for the cognitive deficits induced by neurovascular dysfunction [81].

Nowadays, astrocytes are recognized as key elements for the metabolic supply to neurons by blood vessels. Astrocytes are, in fact, an element of the neurovascular unit, which regulates cerebral blood flow, BBB permeability, neuroimmune responses, and neurovascular remodeling [82]. Astrocytes can regulate cerebral blood flow through their processes by directly interacting with endothelial cells surrounding the brain vasculature. These specialized processes are called astrocytic endfeet [81].

The astrocytic endfoot is a specialized unit that functions to maintain the ionic and osmotic homeostasis of the brain. In transgenic models of cerebral amyloid angiopathy and in hyperhomocysteinemia models of VCI, it has been demonstrated that vascular alteration leads to disruption of the astrocytic endfoot [83,84]. In addition, it was shown that endfoot disruption is subsequent to microglia activation and to the release of proinflammatory cytokines [68,84]. This latter finding suggested the hypothesis that, during inflammatory processes, astrocytes and their endfeet may undergo degeneration because of the release of MMPs [85] which degrade the dystrophin–dystroglycan complex anchoring the endfoot to the basement membrane of the vasculature [65,69,81,85]. Endfeet degeneration, in turn, leads to impaired neurovascular coupling and impaired potassium homeostasis, increasing neuronal excitability and ultimately leading to cognitive deficits [86]. It should also be mentioned that astrocytes, through astrocytic CN/nuclear factor of activated T cell (NFAT), can also release inflammatory cytokines, affecting cognition [87].

Based on these data, the role of astrocytes in VCI-induced cognitive dysfunctions has been increasingly recognized [88]. Several investigations using recent genetic tools now support this notion by showing that inactivating or boosting astroglial function directly affects cognitive abilities [89].

### 4.4. Endothelial Cells and Nitric Oxide

Endothelial cells are part of the neurovascular unit and cover the internal surface of cerebral blood vessels [90]. Many studies have shown that endothelial cells play an important role in VCI. Endothelial cells damaged by oxidative stress contribute to cerebrovascular impairment and neurovascular uncoupling [91]. Beyond that, the link with cognitive dysfunction seems to be due to the effects on the production of nitric oxide (NO) by these cells [92].

NO is a reactive gas secreted in endothelial cells by the endothelial isoform of the enzyme NO synthase, and is tonically released to control systemic vascular tone and neuronal activity in the CNS [93]. In chronic cerebral hypoperfusion models of VCI [94] and in hypertensive rats [95], it has been observed that the bioavailability of endothelium-derived NO is reduced, and that this reduction may be the cause of cognitive deficits. Further support to this hypothesis comes from studies in AD animal models, where a reduction of NO increases the negative effects of amyloid beta (Aβ) on cognition, whereas the administration of NO has a protective effect on Aβ deposition [96].

Many studies are now concentrating on treatments aimed at restoring NO levels at normal conditions in VCI models [92,95,97].

The effects of NO on cognitive processes have been studied, and it has been seen that they are mediated by several mechanisms. NOS is postsynaptically colocalized with *N*-methyl-d-aspartate (NMDA) receptors. After Ca^2+^ influx into postsynaptic neurons, NO acts as a retrograde messenger, providing a positive feedback mechanism to maintain presynaptic glutamate release which binds to various types of postsynaptic NMDA receptors, strengthening hippocampal memory-related processes, such as long-term potentiation (LTP) [93]. NO also regulates other pathways via the post-translational modification (*S*-nitrosylation and 3-nitrotyrosination) of proteins involved in synaptic transmission and intracellular trafficking [98].

At the same time, however, it should be noted that excessive production of NO can have negative effects through the excessive stimulation of NMDA receptors (excitotoxicity), which in normal conditions is counteracted by the same NO through induction of cyclic guanosine monophosphate (cGMP)-mediated signaling [99]. For these reasons, a reduction in NO release by endothelial cells may actually play a very significant role in explaining the association between vascular lesions and cognitive impairment. Other studies are, however, necessary to better define the neuroprotective versus neurotoxic effects of NO in VCI.

### 4.5. Pericytes

Pericytes are mural cells of the neurovascular unit that surround endothelial cells [100]. The main function of pericytes is to regulate the permeability of the BBB and the clearance and phagocytosis of cellular debris [100].

Pericytes have a strong interaction with endothelial cells, forming direct cell–cell contacts, known as “peg-and-socket” contacts [101]. This interaction is essential for both types of cells. Pericytes are essential for the differentiation and survival of endothelial cells. At the same time, the proliferation and migration of pericytes depend on the release of platelet-derived growth factor B (PDGF-B) by endothelial cells [102]. PDGF-B binds to the receptor located in the pericyte membrane and activates signal transduction pathways that include TGF-β [103] and Notch [104]. Therefore, a loss of pericytes can have deleterious consequences on endothelial cells and on BBB permeability [104].

In animal models of neurodegenerative diseases and in post-mortem histological studies, a loss of pericytes has been observed, in particular, in Alzheimer’s disease [105,106]. The mechanism of pericyte loss in neurodegenerative disorders has not been yet elucidated [107]. Preliminary data have suggested that vascular factors, such as hypertension and hyperglycemia, can lead to pericyte loss [108]. Concomitantly, the loss of pericytes also leads to endothelial cell death and can exacerbate vascular dysfunction, causing regression of brain microvessels and giving rise to a condition of chronic hypoxia [109].

In pericyte-deficient transgenic mice induced by manipulation of PDGF-B gene, it has been shown that pericyte degeneration leads to BBB disruption and accumulation, in the brain, of blood-derived products potentially toxic for the neurons and neurovascular unit [110], such as plasmin, thrombin, and fibrin [111]. Pericytes are also involved in Aβ clearance through receptor LRP-1, which binds and internalizes different Aβ species [112]. A loss of brain pericytes may thus lead to reduced Aβ clearance through the LRP-1 degradative pathway, promoting Aβ accumulation and neuronal death. It was also shown that vascular damage caused by pericyte loss is sufficient to induce neurodegeneration, even in absence of Aβ accumulation [105,113].

These data suggest that pericytes may have a prominent role in inducing memory deficits in VCI. Supporting this idea, it was shown in a recent study using a rat model of cerebral small vessel disease that restoration of BBB integrity by infusion of precursor cells of pericyte and endothelial cells inhibits brain atrophy and restores cognitive functions [114].

### 4.6. Autophagy

In eukaryotic cells, autophagy is a fundamental process that degrades and recycles cellular constituents [115]. In the nervous system, autophagy allows nerve cell survival and reparation by clearing abnormally aggregated proteins and damaged cellular organelles, including mitochondria [116]. Autophagy is thus important in a condition of neuronal insult of vascular origin, and it has been demonstrated that it could be induced by oxidative stress [117].

Nonetheless, in a condition of chronic brain hypoperfusion, excessive activation of autophagy can cause cell death (autophagic cell death) [118] through molecular mechanisms involving multiple pathways such as AMP-activated protein kinase (AMPK) or the protein called mammalian target of rapamycin (mTOR) [119].

In rat models of VD, it was shown that excessive autophagy is present in the hippocampus and aggravates neuronal injury [120]. Similarly, in rat models of chronic brain hypoperfusion, the levels of autophagy-related proteins (Beclin-1, light chain 3B, and P62) were found to be increased before the occurrence of cognitive decline [121], suggesting the involvement of autophagy in the pathogenesis of VD. This hypothesis has been confirmed by studies showing neuroprotective and beneficial effects on cognitive function, with treatment aimed at reducing autophagy in these models [122].

There are various proposed molecular mechanisms, which include inhibition of mTOR [123], suppression of autophagy-related proteins, stimulation of vascular endothelial growth factor (VEGF) pathway [124], and reduction of proinflammatory cytokines [125].

Altogether, these data suggest that the suppression of excessive autophagy could have a neuroprotective effect and could be beneficial to preventing VCI. However, it should be noted that suppression of autophagy must be tuned gently during VCI course because beneficial effects of autophagy at the beginning of the condition related to brain hypoperfusion have been reported [126].

### 4.7. Insulin-Like Growth Factor-1

The incidence of VCI in humans increases with age [14]. One of the most interesting endocrine mechanisms connected to age-related cerebrovascular alterations is the decline in circulating insulin-like growth factor 1 (IGF-1) levels, which appears to contribute significantly to vascular aging and age-related cerebrovascular changes [127].

IGF-1 is a single-chain polypeptide widely expressed in brain [127]. IGF-1 is essential for neuroprotection, normal growth, and development [128]. During ontogenesis, IGF-1 exerts its roles on brain development through control of neurotrophic responses and cell signaling [129].

IGF-1 expression is high at young age and declines during aging, and this reduction correlates with cognitive decline in the elderly [130]. IGF-1 regulates cognitive functions by enhancing excitatory synaptic transmission in the CA1 region of the hippocampus [131]. After binding to its receptor (IGF1R), IGF-1 activates important pathways for memory processes, such as PI3K/mTOR/AKT1 (phosphatidylinositol-3 kinase/mammalian target of rapamycin/serine–threonine-specific protein kinase AKT-PKB), and MAPK/ERK (mitogen-activated protein kinase/extracellular signal-regulated kinase) [132].

In addition, IGF-1 induces angiogenesis [133] and neurogenesis [134] in hippocampus. These latter effects are important in the context of VCI pathology. Preclinical studies established a causal link between cognitive decline and microvascular rarefaction in the hippocampus [135]. IGF-1 has important actions on the brain vessels. It has been shown that age-related decline in circulating IGF-1 levels results in functional impairment of the cerebral microvessels [136]. Knockout of the IGF-1 gene produced neurovascular uncoupling in mice [137]. In addition, when hypertension conditions were induced in the same mice, cerebrovascular autoregulatory dysfunctions [138] and microvascular rarefaction in the hippocampus and neocortex [136] were present.

In non-genetic models of VCI (BCVAO), it was shown that IGF-1 and IGF-1 mRNA were downregulated in the hippocampus [139]. Moreover, in human subjects affected by cognitive deficits, it was demonstrated that the reduction of IGF-1 serum levels represents a risk factor for VD [140] and stroke [141].

All these data indicate that IGF-1 may play an important role in maintaining cognitive function and that VCI-induced cognitive are associated with reduction of this peptide. Supporting this notion, IGF-1 has been demonstrated to have potential neuroprotective effects in treating cerebral ischemia. Treatments aimed at restoring IGF-1 brain levels in MCAO rodent models reduced the infarct volume [142] and the rate of apoptosis [143], improving cognitive deficits through the IGF-1/AKT pathway [144].

Notably, peripherally injected IGF-1 can cross the BBB and exert its effect in the CNS [145]. This fact is of considerable interest as a potential treatment for VCI [146]. However, despite the well-recognized positive effects on synaptic function and cognition, the complex role of IGF-1 in vascular and neurodegenerative diseases is still unclear and requires additional research.

## 5. Conclusions

In this review, we analyzed the most important factors contributing to the onset of cognitive deficits in vascular disorders in humans by exploring data from animal models. The animal models of VCI allow understanding of the important molecular mechanisms leading to the cognitive deficits observed in humans. Moreover, these models also allow testing of the various therapeutic strategies based on various experimental hypotheses.

**Figure 1:**
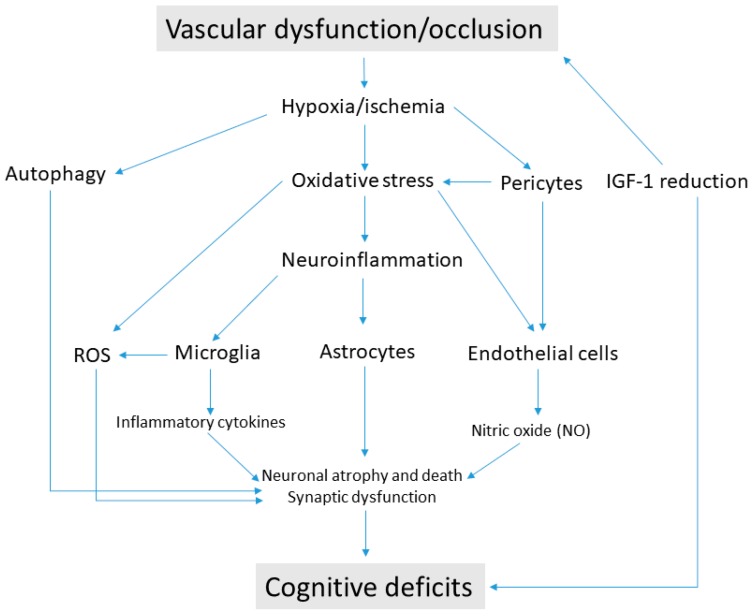
Pathogenic mechanisms causing cognitive deficits in vascular cognitive impairment. Vascular dysfunction/occlusion induces a state of hypoxia/ischemia at the neurovascular unit. The consequent oxidative stress triggers the production of reactive oxygen species (ROS) and sustains the process of neuroinflammation in glial cells. Microglia activation produce proinflammatory cytokines and destruction of astrocyte endfeet contribute to disrupting cytoarchitecture in brain regions involved in cognitive function. Hypoxia/ischemia is also responsible for the loss of pericytes, which cause blood–brain barrier (BBB) and endothelial cell dysfunction. Endothelial cells damaged by oxidative stress cause reduced release of nitric oxide (NO), while excessive autophagy contributes to neuronal damage. The age-related decline of insulin-like growth factor-1 (IGF-1) may be the cause of vascular pathologies from one side, and it may impair synaptic activity from the other side, thus generating cognitive deficits.

VCI may be caused by various mechanisms and metabolic pathways. It is difficult to determine which one is the most important, and the whole picture looks more multifactorial. Certainly, these events are interconnected and may produce cascade effects resulting in cognitive impairment. Initially, a chronic condition of hypoperfusion or hypoxia/ischemia engages a series of reactions at the level of the neurovascular unit, of which the most relevant appear to be oxidative stress and the induction of a neuroinflammatory state. Downstream processes include the activation of the microglia and the release of ROS. Recently, the role of astrocytes has also been greatly emphasized as they are elements of structural and functional support of the neurovascular unit. Astrocytes support the interactions between the vascular and nervous systems through the astrocytic endfeet, which can be destroyed by the release of inflammatory mediators, such as cytokines and ROS, but also by an excessive increase in autophagy. Increased autophagy represents a functional attempt by neurons to recovery from degeneration, but in a chronic condition, it can cause neuronal death implying cognitive deficits. In addition, the activity of endothelial cells surrounding blood vessels may also be affected by oxidative stress, neuroinflammation, and autophagy. In such conditions, these cells decrease the production of NO, a gas of fundamental importance for the activity of hippocampal neurons (LTP) and synaptic plasticity. Loss of pericytes can also exacerbate endothelial cell dysfunction and contribute to generating oxidative stress and regression of brain microvessels. All these alterations can actually be interconnected, as summarized in Figure 1. Therefore, interfering one or more of these factors has led to beneficial effects, at least in laboratory animals. Nonetheless, it should be noted that animal models, especially rodents, cannot effectively represent the complex clinical picture of VCI in humans. Moreover, potential treatments, like those currently available, are aimed at reducing the symptoms but not at curing the causes of VCI.

Studies on IGF-1 factor seem very interesting for a number of reasons: First, a reduction of this factor is associated with the onset of vascular pathologies and, simultaneously, with cognitive decline. Secondly, IGF-1 appears to be able to bypass the BBB and produce beneficial effects on both the vascular and nervous systems. However, although its positive effect on synaptic function are fairly well-recognized, its potential in the prevention and treatment of VCI requires more investigation, possibly using animal species with a more evolved brain (i.e., primates).

## Figures and Tables

**Table 1:** Main rodent models to study vascular cognitive impairment.

Pathology/Risk Factor	Experimental Procedure	Disease Model Name	Species	Reference
Stroke	Surgery	tMCAO, pMCAO	Mice, Rats	[41,42]
Hypertension, stroke	Inbred strain manipulation	SHR/SP	Rats	[43]
Small vessel occlusion	Surgery	tMCAO	Mice, Rats	[44]
Global hypoperfusion		BCAO BCAS		[43,45]
Cerebral/subarachnoid hemorrhage	Autologous blood injection into the brain	SAH	Mice, Rats	[46]
	Collagenase injection into the brain	Collagenase-induced	Mice, Rats	[46]
	Microballoon brain insertion	Microballoon	Mice, Rats	[47]
	Focused laser	Laser-induced	Mice, Rats	[48]
CADASIL	Genetic manipulation	*Notch3* gene mutant	Mice	[41,43]
Hyperhomocysteinemia	Diet-induced	HHcy	Mice, Rats	[49]

Transient middle cerebral artery occlusion (tMCAO); permanent middle cerebral artery occlusion (pMCAO); bilateral carotid artery occlusion (BCAO); bilateral carotid artery stenosis (BCAS); stroke-prone spontaneously hypertensive rats (SHR/SP); subarachnoid hemorrhage model (SAH); cerebral autosomal dominant arteriopathy with subcortical infarcts and leukoencephalopathy (CADASIL); hyperhomocysteinemia (HHcy).
